# Forecasting drug utilization and expenditure: ten years of experience in Stockholm

**DOI:** 10.1186/s12913-020-05170-0

**Published:** 2020-05-11

**Authors:** Love Linnér, Irene Eriksson, Marie Persson, Björn Wettermark

**Affiliations:** 1 Public Healthcare Services Committee, Region Stockholm, Stockholm, Sweden; 2 Department of Medicine Solna, Centre for Pharmacoepidemiology, Karolinska Institutet, Stockholm, Sweden

**Keywords:** Pharmaceutical expenditure, Drug utilization, Forecasting

## Abstract

**Background:**

Operating under constrained budgets, payers and providers globally face challenges in enabling appropriate and sustainable access to new medicines. Among payer initiatives aiming to improve preparedness of healthcare systems for the introduction of new medicines, drug utilization and expenditure forecasting has played an increasingly important role. This study aims to describe the forecasting model used in Region Stockholm and to evaluate the accuracy of the forecasts produced over the past decade.

**Methods:**

In this repeated cross-sectional study, we compared the predicted pharmaceutical expenditure with actual expenditure during the entire available follow-up period (2007–2018) both for overall drug utilization and for individual therapeutic groups. All analyses were based on pharmaceutical expenditure data that include medicines used in hospitals and dispensed prescription medicines for all residents of the region.

**Results:**

According to the forecasts, the total pharmaceutical expenditure was estimated to increase between 2 and 8% annually. Our analyses showed that the accuracy of these forecasts varied over the years with a mean absolute error of 1.9 percentage points. Forecasts for the same year were more accurate than forecasts for the next year. The accuracy of forecasts also differed across the therapeutic areas. Factors influencing the accuracy of forecasting included the timing of the introduction of both new medicines and generics, the rate of uptake of new medicines, and sudden changes in reimbursement policies.

**Conclusions:**

Based on the analyses of all forecasting reports produced since the model was established in Stockholm in the late 2000s, we demonstrated that it is feasible to forecast pharmaceutical expenditure with a reasonable accuracy. A number of factors influencing the accuracy of forecasting were also identified. If forecasting is used to provide data for decisions on budget allocation and agreements between payers and providers, we advise to update the forecast as close as possible prior to the decision date.

## Background

Over the past decades, pharmaceutical expenditure has been rising in many countries [1–3]. This growth has been attributed to a number of factors including ageing populations, increasing patient expectations, as well as the introduction of new and more expensive medicines [4, 5]. In parallel, payers have been implementing a range of initiatives to promote rational use of medicines and get a better control of the budgets [5, 6]. Examples of such initiatives include activities to facilitate the prescribing and dispensing of generics, measures to limit the use of new medicines of uncertain value, treatment guidelines, economic incentives to prescribers, and various reimbursement strategies [5–7].

Various approaches to managed introduction of new medicines have also been established to enable cost-effective and evidence-based use, particularly given the uncertainties about the use and outcomes in routine clinical practice [4, 5, 8]. A functional managed introduction process requires a number of proactive steps along the timeline of the introduction of a new medicine [8, 9]. First, emerging new health technologies need to be identified prior to marketing authorization. This task is typically fulfilled by horizon scanning systems [9]. Next, drug utilization and expenditure forecasts should provide decision makers with necessary information to allocate resources and set up activities promoting the rational uptake and use of new and established medicines [10]. Both horizon scanning and forecasting have been adopted as tools by many payers internationally.

In Stockholm, forecasting has been used for more than a decade as part of a regional process for managed introduction of new medicines [10]. However, despite that forecasts have been made for more than a decade, assessment of the accuracy of our predictions has been limited. Similarly, even though forecasting has been used by many other payers internationally, there are few studies on forecasting of pharmaceutical expenditure published to date. Some of these studies are focused on the forecasting methods [11–14] and some presented projections of pharmaceutical expenditure [15–19] including comprehensive approaches to cover all therapeutic areas [20, 21]. The accuracy of forecasting has also been evaluated [22, 23]. One of these studies assessed the accuracy of analysts’ estimates of peak sales of new medicines launched from 2002 to 2011 [22]. The study found that most consensus estimates provided by analysts were wrong, often substantially, with the sales of central nervous system and cardiovascular medicines being overestimated and the sales of oncology medicines being underestimated. Another recent study also assessed the accuracy of the US forecasts of pharmaceutical expenditure published annually in the American Journal of Health-System Pharmacy and found that the forecasts were reasonably accurate in predicting the growth in expenditure [23].

The objectives of our study are to describe the model that has been used for forecasting drug utilization and expenditure in Region Stockholm and to evaluate the accuracy of the model’s predictions since its inception until 2018. In addition, the current forecast for 2019–2020 is also presented.

## Methods

### Study design

In this repeated cross-sectional study, we compared the predicted pharmaceutical expenditure with actual expenditure during the entire available follow-up period (2007–2018). Analyses were conducted for total pharmaceutical expenditure to assess whether the accuracy improved over time. Furthermore, a stratification by therapeutic group was performed to identify and examine the factors influencing the accuracy of forecasting.

### Setting

This study was conducted in Stockholm, which is the largest healthcare region of Sweden. Region Stockholm has a population of 2.3 million living in 26 municipalities, including the city of Stockholm, urban area municipalities, large rural areas, and a sparsely populated archipelago.

The Swedish healthcare system is decentralized and regions are largely responsible for decision making and provision of healthcare services, including financing of outpatient and inpatient medicines. Healthcare services are financed by local taxes and supplemented by central government grants and patient copayments [24].

A number of national reforms were implemented during the 2000s to promote rational use of both new and established medicines [25, 26], including the development of a process to support rational introduction of new medicines [9]. This process comprises a number of steps, including horizon scanning to identify new medicines as well as forecasting of pharmaceutical expenditure. A recent study found that the Swedish horizon scanning system was able to identify and prioritize all innovative medicines that went on to have substantial economic impact [27]. Stockholm’s forecasting model relies on horizon scanning for information on new medicines expected to be marketed in the coming years [10].

### Forecasting model

**Fig. 1:**
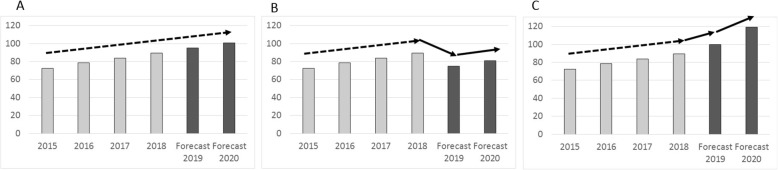
Illustration of the forecasting model. **a** Actual expenditure during 4 years with predicted growth for 2 years based on linear regression. **b** Adjusted growth in expenditure to account for a patent expiry and introduction of generics. **c** Adjusted growth in expenditure to account for a new medicine to be launched

The annual forecast has normally been published as a report in the beginning of each year, covering the coming 2 years. For example, if the report was published in March 2016 it presented the forecast for 2016 (same year forecast) and 2017 (next year forecast). In short, the forecast has been performed as follows. First, all medicines are divided into individual therapeutic groups primarily based on the second or third level of Anatomical Therapeutic Chemical (ATC) Classification System. The definition of a therapeutic group is tailored when necessary, for example, the multiple sclerosis therapeutic group includes all relevant medicines that are spread across several ATC groups. Then, for each individual therapeutic group, a linear regression model is fitted to a time series of drug utilization data from the previous 4 years and crude predictions for the current and next year are based on a linear extrapolation (Fig. 1).

For each therapeutic group the automated prediction is adjusted for factors likely to increase or decrease future drug utilization and expenditure, such as patent expiries, new medicines to be launched, new indications, or new treatment guidelines from national agencies and the regional Drug and Therapeutics Committee. The decision to make an adjustment is informed by input from pharmacists, clinical pharmacologists, and clinical experts from the regional Drug and Therapeutics Committee. Given that the ageing of the population, population growth, and financial incentives used to steer the prescribing are already accounted for when drug utilization and expenditure trends are derived, no additional adjustment for these factors is made. All forecasts are based on pharmaceutical expenditure data that include all medicines used in hospitals and all dispensed prescription medicines in ambulatory care (reimbursed expenditure and copayment). A detailed description of the forecasting model has been published elsewhere [10]. Pharmaceutical expenditure is reported in the official currency of Sweden (Swedish Krona, SEK). The exchange rate as of August 2019 is Euro 1.00 = SEK 10.70.

### Data sources

Pharmaceutical expenditure data and the information published in the forecasting reports were collected to assess the forecasting accuracy. For the analysis of the overall accuracy we retrieved all forecasting reports produced between 2007 and 2018. These reports include both the forecasted expenditure for the two coming years (same and next year) as well as aggregate pharmaceutical expenditure data for the four previous years. All pharmaceutical expenditure data (i.e. medicines used in hospitals and dispensed prescription medicines for all residents of the region) were extracted from the data warehouse (VAL) that is owned and operated by Region Stockholm. Overall, the content of the VAL databases ranges from detailed information on primary care visits and use of medicines to migration dates to and from the region. All healthcare providers that are contracted by Region Stockholm regularly submit information and the databases are generally updated on a monthly basis.

**Fig. 2:**
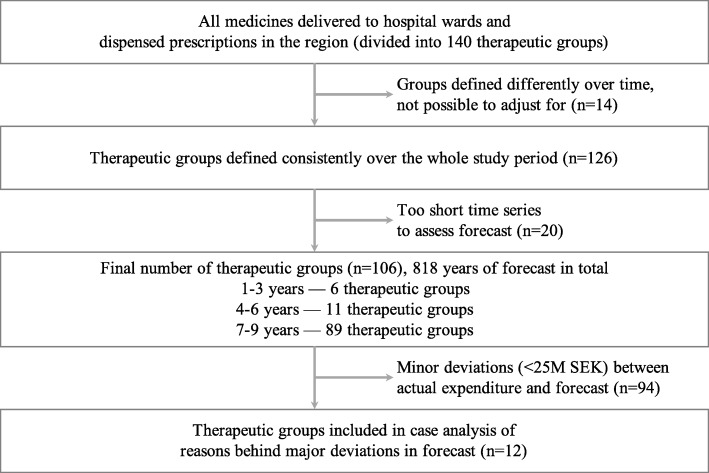
Selection of individual therapeutic groups

For the analysis of the individual therapeutic groups, we first retrieved the original datasets used in the respective forecasting models. Data from the forecasts from 2009 to 2018 were available. Next, we assessed the consistency of the definition of each therapeutic group because some of the definitions were changed over the years. For example, in 2014, the therapeutic group ‘antivirals’ was divided into several subgroups in response to the introduction of the new medicines for hepatitis C. To derive continuous time series for as many therapeutic groups as possible, some of the groups that were at some point divided (such as the earlier mentioned ‘antivirals’) would be combined again for the purposes of our analyses. If the changes to the therapeutic group definition were too substantial and regrouping therefore was not possible, then these therapeutic groups were excluded from further analyses—either for the entire period or for a number of sequential years (Fig. 2). Overall, of the 140 therapeutic groups available for analysis, 14 were completely excluded due to the inconsistency of the definition used over the years.

As described earlier, each forecasting report provides predictions for 2 years. Therefore, for each calendar year, there are two forecasts available—first is the forecast that was produced in the previous year and second is the forecast that was produced in the beginning of the year—as well as the actual expenditure data for this year. Twenty individual therapeutic groups had time series that were too short to include these three datapoints (two estimates from the forecasts and the actual expenditure data). Therefore, upon excluding these, 106 groups remained in the analyses of forecasting accuracy across the individual therapeutic groups.

### Statistical analyses

All data management and analyses were conducted using R (www.r-project.org) and the R package Plotly was used for creating the figures (Plotly Technologies Inc., www.plot.ly).

### Accuracy of overall forecast

We calculated the error (measured in percentage points) in the overall forecast. Percentage change in total actual expenditure from the previous year to the current year was compared to the forecasted percentage change in expenditure in the two forecasts (same year forecast and previous year forecast). Correlation of these values was assessed in a linear regression analysis. If at least one of the regressions (same year or previous year forecast) showed a significant relationship, the difference between coefficients for the forecasts was evaluated through linear regression with an interaction term for the type of forecast. Difference between forecasted and actual expenditure over time was also analyzed using linear regression.

### Accuracy of forecast for individual therapeutic groups

For each individual therapeutic group and year actual change in expenditure from the previous year to the current year was compared to the forecasted change in expenditure in the two forecasts (same year forecast and previous year forecast). In the regression model each yearly forecast for each group was assumed to be independent from forecasts for the same group in other years (no group effects over time). If at least one of the regressions (same year or previous year forecast) showed a significant relationship, the difference between coefficients for the forecasts was evaluated through linear regression with an interaction term for the type of forecast. An arbitrary error threshold (absolute difference between actual and predicted change in expenditure) of SEK 25 million (M) was used to select the individual therapeutic groups for further analyses of the factors potentially influencing the accuracy of our forecasts.

## Results

### Overall forecast

**Fig. 3:**
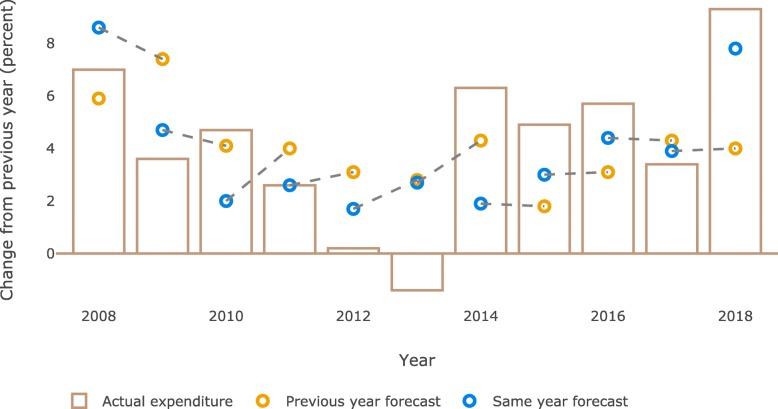
Predicted and actual change in pharmaceutical expenditure (%). Dashed line indicates the forecasts published within the same forecasting report (i.e. same and next year forecast)

According to the forecasts, the total pharmaceutical expenditure was estimated to increase between 2 and 8% annually. In 2011, the actual expenditure increased by exactly the same amount as forecasted at the start of that year. The largest errors for the same year forecast were observed in 2013 and 2014 (Fig. 3). Moreover, we found that for the same year forecast the error was less than 1.5 percentage point in 5 out of 10 forecasts (for two out of these five the error was less than one percentage point). The mean error was 1.9 percentage point (standard deviation: 1.3 percentage point).

Regression analyses of predicted and actual change in total expenditure indicated that the forecasts done for the same year were closer to the actual expenditure than the forecasts done in the previous year (same year: Coefficient = 0.827, R^2^ = 0.339, *p* < 0.05; previous year: Coefficient = 0.48, R^2^ = − 0.05, *p* > 0.05). No significant difference between the coefficients of the same year and previous year forecasts was observed. Accuracy for the same and previous year forecasts over time revealed no significant trend (Coefficient_same year_ = 0.00; Coefficient_previous year_ = 0.16).

### Forecast for individual therapeutic groups

**Fig. 4:**
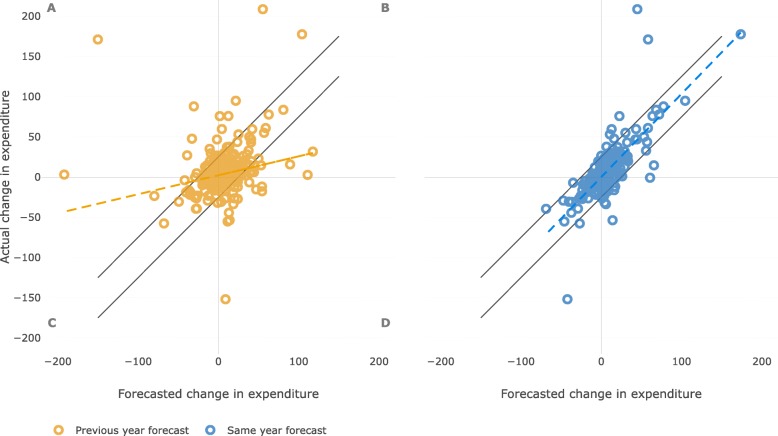
Predicted and actual change in pharmaceutical expenditure (M SEK) for individual therapeutic groups. Dashed lines represent linear regression for predicted and actual change. Black lines represent the threshold for further analyses (SEK 25 M error in forecast). Letters in quadrants indicate A. predicted decrease but actual increase, B. predicted and actual increase, C. predicted and actual decrease, and D. predicted increase but actual decrease in expenditure

One hundred and six individual therapeutic groups were included in the analysis. For each of these groups forecasts for several years were available with a total number of 818 group–year combinations available for analyses (Fig. 4). Our analyses showed differences between the accuracy of forecasts done in the same and in the previous year (same year: Coefficient = 1.03, R^2^ = 0.58, *p* < 0.001; previous year: Coefficient = 0.24, R^2^ = 0.05, p < 0.001). The coefficients of the same and the previous year forecasts were significantly different (p < 0.001).

**Fig. 5:**
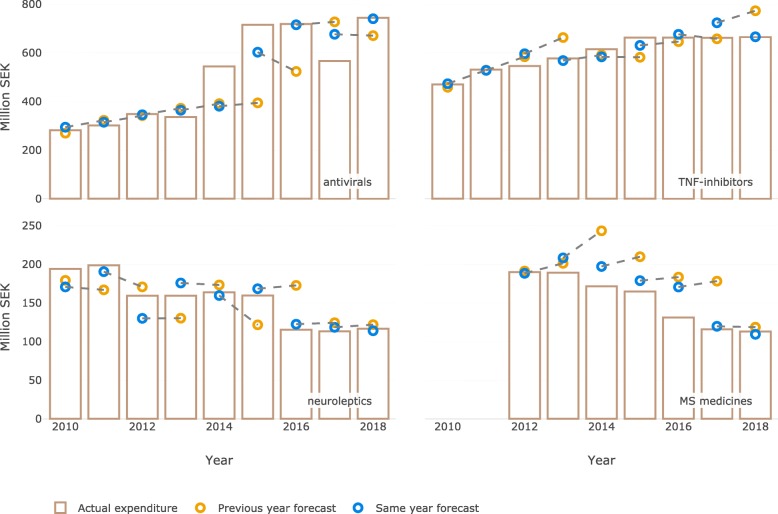
Individual therapeutic groups with major errors in the same year forecast. Predicted and actual change in expenditure (M SEK). Dashed line indicates the forecasts published within the same forecasting report (i.e. same and next year forecast)

Based on the error threshold of SEK 25 M we identified twelve groups with major differences between the actual and predicted expenditure occurring at some point in time in the past decade (Table 1). The actual and predicted expenditure over time for four of these groups is illustrated in Fig. 5.

**Table 1:** The list of individual therapeutic groups with the difference between predicted and actual change in expenditure exceeding SEK 25 M

Therapeutic group	Difference between the forecasted yearly change and the actual change (M SEK)	Year when the difference occurred	Direction of the difference (underestimated or overestimated)
Antivirals^a^	164	2014	underestimated
TNF-inhibitors^a^	62	2017	overestimated
Oncology: monoclonal antibodies	47	2014	underestimated
Immunosuppressants (excluding TNF-inhibitors)	68	2010	overestimated
Oncology: kinase inhibitors	42	2016	underestimated
Coagulation factors	32	2015	overestimated
Neuroleptics^a^	29	2012	underestimated
Multiple sclerosis medicines^a^	39	2016	overestimated
Perfusion solutions	36	2013	overestimated
Angiotensin receptor blockers	31	2013	overestimated
Nonsteroidal anti-inflammatory drugs	27	2010	overestimated
Anti-dementia drugs	28	2012	underestimated

^a^Additional information is presented in Fig. 5

### Current forecast for 2019 and 2020

The most recent forecast of pharmaceutical expenditure in Region Stockholm was published in May 2019 and it covers 2 years: 2019 and 2020 (Table 2). The total pharmaceutical expenditure in the region (hospital use and prescription medicines excluding patient copayment) was estimated to increase from SEK 8.1 billion (B) in 2018 to 8.7 billion and 9.4 billion in 2019 and 2020, respectively. This corresponds to annual increases of 7.0 and 8.0%, respectively.

**Table 2:** Forecast (overall) for pharmaceutical expenditure (M SEK) in Region Stockholm (data as of May 2019)

	Year			
	2017	2018	2019 (forecast)	2020 (forecast)
Pharmaceutical expenditure	7163	7574	8017	8656
Rebates/risk-sharing agreements	210	565	694	749

The current forecast takes into account the rebates and risk-sharing agreements for prescription medicines that in recent years have become more frequent in Sweden. If such rebates are adjusted for in the forecast, then the estimate for the annual increase changes for 2019 (estimated 5.9% increase), however it remains the same for 2020 (estimated 8.0% increase).

**Fig. 6:**
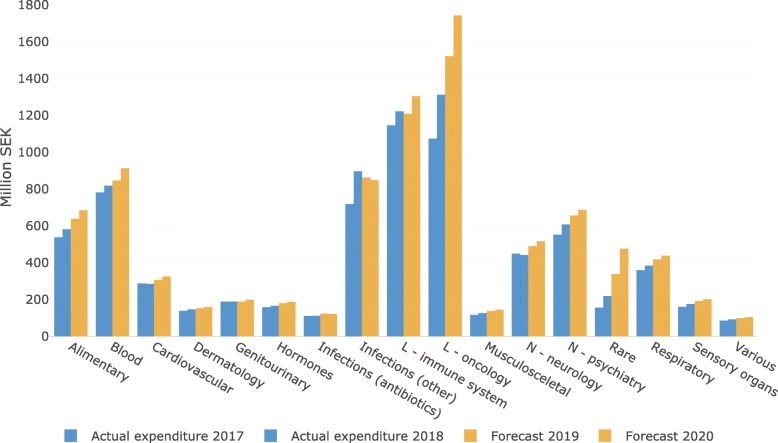
Forecast (individual therapeutic groups) for pharmaceutical expenditure (M SEK) in Region Stockholm (data as of May 2019). ATC 1st level groups. ATC groups J, L, and N are divided in subgroups

Overall, the most pronounced growth in pharmaceutical expenditure is expected for cancer medicines as well as medicines used for very rare diseases (Fig. 6). It can also be seen that in the recent years the actual expenditure for antivirals (specifically medicines for HIV and hepatitis C) have fluctuated greatly as a result of measures taken to facilitate their managed introduction. In the current forecast the expenditure for antivirals is expected to gradually decrease due to an increased competition between the available products as well as a decreasing number of patients with hepatitis C who remain to be treated. The use of new anticoagulants and new diabetes medicines is also expected to continue growing with a corresponding increase in expenditure. Given the recent inclusion of new medicines for treatment of cystic fibrosis, migraine, and breast cancer in the national pharmaceutical reimbursement scheme, the expenditure in these therapeutic areas will also grow. At the same time, we anticipate that the expenditure on TNF-inhibitors will decrease due to the biosimilar competition and competition from other new medicines such as JAK inhibitors. (Fig. 6).

## Discussion

This study examined the forecasting conducted in Stockholm over the past decade. We found that the forecasting accuracy varied over the years with a mean error of 1.9 percentage points. For five out of 10 years available for analysis the difference between the forecast (same year) and the actual expenditure was less than 1.5 percentage points. The largest discrepancies in the Stockholm forecasts compared to the actual expenditure were observed in 2013 and 2014, with more than 4 percentage points overestimation and 4 percentage points underestimation, respectively. For 2013 the forecast overestimated the expenditure in part due to misjudging the impact and timing of patent expiries, but also due to a change in how hospital drug expenditure was calculated. For 2014 we underestimated the increase in expenditure primarily due to the very rapid uptake of medicines for hepatitis C in the end of 2014.

The accuracy of our forecasts neither increased nor decreased over the years. Therefore, our perceived accumulated knowledge and experience in forecasting does not appear to translate into an improvement in accuracy. This may be partly explained by the fact that information on timing of future patent expiries and introduction of new medicines is crucial for the accuracy of forecasting. The availability and reliability of this information however is limited.

Our analyses of forecasting accuracy for individual therapeutic groups showed that the majority of our predictions were reasonably accurate. Major discrepancies were however identified over the years for several important therapeutic groups. In the original forecasts patent expiries and subsequent introduction of generics and biosimilars as well as changes in price or reimbursement status were among the key factors expected to decrease expenditure that were modelled into the forecast [10]. The major factor expected to increase expenditure was the introduction of new medicines as well as the use of medicines in new indications. Factors such as treatment recommendations, introduction of incentives or budgets, and major structural changes in healthcare provision, organization, and reimbursement were considered as having a variable impact and were modelled individually for each therapeutic area [10]. The major errors that we identified in our forecasts were indeed largely explained by the above listed factors.

Patent expiries and the subsequent introduction of generics have a big impact on drug prices in Sweden with the main effects normally observed after a few months following launch of the generic alternatives [28]. Therefore, the precision of our estimate of the timing when generics become available to a large degree affects the accuracy of our forecast. While most of such estimates were rather precise, we however in some instances incorrectly estimated the timing of patent expiry or the level of subsequent price reduction. For example, in our forecast for 2012 the impact of patient expiries on antipsychotic drug expenditure was overestimated by SEK 29 M (Fig. 5). The forecast took into consideration that both quetiapine and olanzapine were going off patent. However, it did not account for the increasing use of still patented long-acting formulation of quetiapine.

It has also been challenging to forecast the introduction of biosimilars the availability of which increased considerably over the past decade. While the debate on the effectiveness and safety of biosimilars compared to originator biologics affected the uptake early on, the concerns subsided over the years. Now, biosimilars are seen as an important treatment alternative that comes at a lower cost. Still, in Sweden, the biosimilars are not deemed interchangeable at the pharmacy and the uptake largely depends on the prescriber preferences. Providing an accurate forecast of the biosimilars uptake is therefore still a challenge. For example, in our forecast for 2017 we underestimated the impact of biosimilar competition for etanercept on the total TNF-inhibitor expenditure because the prescribers were more willing to prescribe biosimilars than we anticipated. In addition, new mechanisms in the national pharmaceutical reimbursement process facilitated decreases in list prices. Overall, there are variations in policies on biosimilars across countries, providing an opportunity for cross-national comparison studies examining the impact of these policies on rational introduction of biosimilars [29, 30].

Introduction of new medicines is a key factor considered when forecasting pharmaceutical expenditure. The introduction of many new medicines was adequately forecasted during the study period. Most of the new medicines had a gradual uptake which is easier to forecast. However, new medicines for hepatitis C were introduced very rapidly in Sweden, due to the existing policies on the use of medicines in serious contagious diseases [31]. The forecast therefore underestimated the expenditure on new hepatitis C treatments both in the first and second year after their introduction on the Swedish market. Conversely, there were instances when the forecast predicted the growth in expenditure that never materialized. This can be exemplified by the forecast made for the multiple sclerosis medicines. While many new medicines were introduced during the study period only two of these had a noticeable uptake (fingolimod and dimethyl fumarate). Instead, from 2013 onward the off-label use of rituximab increased rapidly [32]. The forecast for 2019 and 2020 also accounted for the introduction of advanced therapy products such as gene therapy for treatment of hematological cancers and rare inherited disorders. Such specialist treatments however would only be administered in selected few clinics in Sweden thus currently it is unclear how to forecast expenditure for these new treatment options at a regional level.

Changes in prices and reimbursement status have an immediate impact on expenditure. These were difficult to identify more than a year in advance. In 2013, for example, a major change in hospital supply margins was made resulting in the decrease in expenditure for certain products used in hospitals such as perfusion solutions. In ambulatory care, the impact of the reimbursement reviews for antiepileptic medicines (conducted in 2012) and asthma inhalation medicines (conducted in 2015) was anticipated. However, the coming decrease in expenditure for TNF-inhibitors due to a similar reimbursement review was missed in the forecast. Moreover, a political decision to eliminate copayment for all medicines prescribed to children was suddenly implemented in 2016. This resulted in changes in reimbursed expenditure for medicines commonly used among children and adolescents (e.g. antibiotics, antihistamines, emollients, and asthma medicines). However, a recent study focusing on the use of asthma medicines in children showed that this decision had limited impact on the utilization patterns [33].

In recent years the use of confidential rebates and risk-sharing agreements for prescribed medicines has increased [34]. Given their direct impact on the total net pharmaceutical expenditure, rebates were included in the forecast for 2019 and 2020. However, it is difficult to foresee which new risk-sharing agreements will be implemented in the near future as such agreements are dependent on decisions made by multiple stakeholders. Rebates and risk-sharing agreements therefore add another level of complexity in forecasting.

Our assessment of both the overall forecast and the forecast for individual therapeutic groups clearly demonstrated that forecasts for the same year were more accurate than the forecasts for the next year. This is of course expected, but the magnitude of the decrease in accuracy for the next year forecast nonetheless surprised us. We are not aware of any other study describing the impact of the forecasting horizon on the accuracy of predicted pharmaceutical expenditure. A forecast is only valuable if it provides a reasonably accurate estimate of the future pharmaceutical expenditure. Therefore, when planning forecasting activities, a balance between the forecasting horizon and adequate level of precision needs to be found.

Our study has several strengths. First, our analyses are based on more than 10 years of complete pharmaceutical expenditure data including both medicines used in hospitals and dispensed prescription medicines in ambulatory care for all residents of the region. Second, it was possible to assess the impact of the forecasting horizon on the accuracy of the forecast. Third, the original forecasting reports contained detailed comments provided by pharmacists, clinical pharmacologists, and clinical experts from the regional Drug and Therapeutics Committee. This helped understand the rationale for the predicted changes in each therapeutic area and explore the possible factors influencing the accuracy of our forecasts.

Our study however could have been more informative if we could also provide information on the predicted and actual number of patients treated. While it is possible to estimate the number of users of prescribed outpatient medicines, such information is currently not available for the medicines administered in hospitals. Also, due to the inconsistency of used definitions for a number of therapeutic groups, these groups had to be excluded from the analyses of the forecast for individual therapeutic groups. Finally, the increasing use of confidential rebates from 2015 onward makes the interpretation of the forecast more complex.

## Conclusions

The key findings of this study can be summarized as follows. First, it is possible to forecast pharmaceutical expenditure with a reasonable accuracy. However, the pharmaceutical expenditure is influenced by many factors and the pharmaceutical market itself changes rapidly. The involvement of objective clinical experts therefore is necessary to keep abreast of the recent developments across therapeutic areas. Moreover, proactive engagement with both regulatory bodies and pricing and reimbursement agencies can help minimize the risk of missing important events. Second, if forecasting is used to provide data for decisions on budget allocation and agreements between payers and providers, it is wise to update the forecast as close as possible prior to the decision date. Third, there appears to be no added value in developing forecasts of pharmaceutical expenditure with a horizon longer than 2 years because the methods and data available do not seem to provide accurate predictions.

## Data Availability

The data that support the findings of this study are available from Region Stockholm but restrictions apply to the availability of these data, which were used under license for the current study, and so are not publicly available. The datasets analyzed are however available from the authors upon reasonable request and with permission of Region Stockholm.

## Abbreviations

ATC: Anatomical therapeutic chemical
SEK: Swedish krona
M: Million
B: Billion
